# Early and Efficient Detection of *Mycobacterium tuberculosis* in Sputum by Microscopic Observation of Broth Cultures

**DOI:** 10.1371/journal.pone.0057527

**Published:** 2013-02-28

**Authors:** Benson R. Kidenya, Rodrick Kabangila, Robert N. Peck, Stephen E. Mshana, Lauren E. Webster, Serena P. Koenig, Warren D. Johnson, Daniel W. Fitzgerald

**Affiliations:** 1 Department of Biochemistry and Molecular Biology, School of Medicine, Catholic University of Health and Allied Sciences, Mwanza, Tanzania; 2 Faculty of Graduate School, Weill Cornell Medical College, New York, New York, United States of America; 3 Department of Internal Medicine, School of Medicine, Catholic University of Health and Allied Sciences, Mwanza, Tanzania; 4 Department of Microbiology and Immunology, School of Medicine, Catholic University of Health and Allied Sciences, Mwanza, Tanzania; 5 Center for Global Health, Division of Infectious Diseases, Weill Cornell Medical College, New York, New York, United States of America; 6 Division of Global Health Equity, Brigham and Women’s Hospital, Harvard Medical School, Boston, Massachusetts, United States of America; McGill University, Canada

## Abstract

Early, efficient and inexpensive methods for the detection of pulmonary tuberculosis are urgently needed for effective patient management as well as to interrupt transmission. These methods to detect *M. tuberculosis* in a timely and affordable way are not yet widely available in resource-limited settings. In a developing-country setting, we prospectively evaluated two methods for culturing and detecting *M. tuberculosis* in sputum. Sputum samples were cultured in liquid assay (micro broth culture) in microplate wells and growth was detected by microscopic observation, or in Löwenstein–Jensen (LJ) solid media where growth was detected by visual inspection for colonies. Sputum samples were collected from 321 tuberculosis (TB) suspects attending Bugando Medical Centre, in Mwanza, Tanzania, and were cultured in parallel. Pulmonary tuberculosis cases were diagnosed using the American Thoracic Society diagnostic standards. There were a total of 200 (62.3%) pulmonary tuberculosis cases. Liquid assay with microscopic detection detected a significantly higher proportion of cases than LJ solid culture: 89.0% (95% confidence interval [CI], 84.7% to 93.3%) versus 77.0% (95% CI, 71.2% to 82.8%) (*p* = 0.0007). The median turn around time to diagnose tuberculosis was significantly shorter for micro broth culture than for the LJ solid culture, 9 days (interquartile range [IQR] 7–13), versus 21 days (IQR 14–28) (*p*<0.0001). The cost for micro broth culture (labor inclusive) in our study was US $4.56 per sample, versus US $11.35 per sample for the LJ solid culture. The liquid assay (micro broth culture) is an early, feasible, and inexpensive method for detection of pulmonary tuberculosis in resource limited settings.

## Introduction

Tuberculosis (TB) is a disease of poverty; it continues to be a leading cause of morbidity and mortality in developing countries [1], [2], [3], [4], [5], [6]. Global efforts for TB control, especially in resource limited settings, are being challenged by the lack of rapid, reliable and inexpensive techniques for the detection of *M. tuberculosis* [7], [8], [9], [10]. Results from the conventional culture detection methods come too late to influence a timely decision on patient management. Early identification is the key in both patient management and controlling transmission of *M. tuberculosis* [8], [11]. Therefore, more early, inexpensive and reliable tests are urgently needed [7], [12], [13].

Microscopic observation of broth cultures is a low-technology but rapid testing method for detection of tuberculosis [8], [11], [12], [13], [14], [15]. When coupled with drug sensitivity testing, this assay has been called Microscopic Observation of Drug Sensitivity (MODS). Microscopic observation of broth cultures requires no special equipment or technology other than an inverted light microscope to visualize the characteristic cording growth of *M. tuberculosis* through the underside of the micro broth culture plates and has been reported to cost less than US$5.00 per sample [11], [16]. The inverted light microscope, as opposed to a simple microscope, is not a usual standard lab equipment and few labs have this equipment, so evaluation such as this is actually ideal. Therefore we undertook this study in a resource limited setting to operationally evaluate the performance of microscopic observation of broth cultures in detecting *M. tuberculosis* and to determine the feasibility of establishing this assay at a hospital in an underserved area.

## Materials and Methods

### Study Design

This was a cross sectional study in which the sample size was calculated using Buderer’s formula [17] to determine the sensitivity and specificity of 95% with absolute precision of less than 5% at 95% confidence interval [CI] with the prevalence of TB among TB suspects of 60% [18]. We cultured sputa from 321 consecutive suspected tuberculosis patients using both standard LJ solid media and liquid media in microplates. Growth was detected on LJ solid media by visual inspection for colonies. Growth was detected in the liquid broth in microplate wells by microscopic observation. We compared the sensitivity, specificity, contamination rates, time to results, and cost of the two tests.

### Study Patients and Setting

This study was conducted at Bugando Medical Centre, Mwanza, Tanzania, from October 2010 to July 2011. Bugando Medical Centre (BMC) is a 900-bed tertiary hospital serving a population of over 10 million people from six regions in Northwestern Tanzania. According to the 2011 Global TB Control Report, the estimated prevalence of TB in Tanzania in 2010 was 0.15% [19]. Patients who presented with symptoms and signs suggestive of tuberculosis were serially enrolled into the study by physicians. For inclusion, patients had to present with one or more constitutional symptoms (cough, fever, weight loss, night sweats, hemoptysis) and be treatment naive for tuberculosis drugs. Criteria for exclusion were age less than 12 years, inability to produce sputum and inability or unwillingness to give written informed consent. The minimum acceptable volume of sputum was 2 ml; however, patients producing less than 2 ml were not immediately excluded but were encouraged to collect more, exceeding the 2 ml minimum. We obtained the informed written consent from the parents, caretakers, or guardians on the behalf of the minors/children participants involved in our study. Adult participants provided their written informed consent to participate in this study. Study protocol and consent forms were reviewed and approved by both the Catholic University of Health and Allied Sciences (CUHAS)/BMC joint ethics review board and the institutional review board of Weill Cornell Medical College, New York.

### Definition of Tuberculosis Cases

Our case definition of tuberculosis is based upon the definition of the American Thoracic Society (ATS), and has been used in our prior reports [20], [21], [22], [23]. We require two of the following three criteria: (i) symptoms of tuberculosis (cough, fever, night sweats, etc.); (ii) a chest radiograph independently interpreted as highly suggestive of tuberculosis; (iii) microbiological confirmation of tuberculosis by detection of acid fast bacilli (AFB) by Ziehl- Neelsen Staining or positive solid or liquid *M. tuberculosis* culture or a pathology specimen with characteristic granuloma formation and AFB. For patients without microbiological confirmation, we require a clinical response to antituberculosis medications. These patients were followed up by physicians for TB treatment outcome. Furthermore, physicians were responsible for determining ATS TB diagnosis and interpreting the chest radiograph. There was a blinding between the clinical determination and chest X-ray reading. Likewise, the lab staff were blinded to clinical data. Experienced laboratory technicians performed the culture examinations and were practically trained before the study. Prior to study commencement, the lab technicians’ skills were tested for competence.

### HIV Diagnosis

Patients were counseled and tested for HIV testing according to the Tanzanian National HIV algorithms which recommend a rapid test qualitative immunoassay. We used Determine (Alere Medical Company LTD, Japan) followed by UNGOLD (Trinity Biotech plc, Bray, Ireland) for participants who had positive results on Determin. All the tests were conducted according to the standards provided by the manufacturers [24].

### Laboratory Methods

#### Sample collection and processing

For the current study we compared the performance of the two culture techniques on a single patient sample. Patients with suspected tuberculosis submitted an early morning sputum to the hospital TB laboratory for routine culture by Löwenstein–Jensen (LJ) media and culture in liquid broth prior to initiation of antituberculosis therapy. The sputum was decontaminated according to the standard sodium hydroxide–*N*-acetyl-L-cysteine (NALC) method [25]. Following centrifugation for 15 minutes at 4,000 × *g*, the pellet was then re-suspended into 2 ml of 7H9 broth. After decontamination, each processed sample was divided into 2 aliquots that were used for parallel LJ and liquid broth culture [7], [8].

#### Growth detection using LJ medium

The decontaminated sample (100 µl) was inoculated on two tubes of LJ solid media made by using standard procedure [26]. To prepare the LJ media, first a mineral salt solution was created composed of 2.4 g potassium dihydrogen phosphate anhydrous (KH_2_PO_4_), 0.24 g magnesium sulphate anhydrous, 0.6 g magnesium citrate, 3.6 g asparagine, 12 ml glycerol (reagent grade), 20 ml malachite green, 2% solution (made of 2.0 g malachite green dye 2.0 g and 100 ml distilled water). The ingredients were dissolved in distilled water in order listed, then cooled to room temperature and refrigerated. Then 600 ml of the mineral salt solution was mixed with 1000 ml of homogenized eggs. The complete egg medium was distributed in sterile, tight-capped universal containers in 6–8 ml volumes and immediately positioned in an inspissator in a slanted position for 50 minutes at 85°C. The LJ medium was then stored in a refrigerator for up to 4 weeks [26]. Each medium slant was properly labeled with the sample number and date of inoculation. The reference strain, ATCC 27294 H37Rv *M. tuberculosis* from Makerere University TB reference Laboratory, Uganda, was also included in each test batch as a quality control for culture. The cultures were incubated at 37°C and inspected first after 48 hours and then examined once weekly from week 1 through week 8 until growth of colonies was observed. Each isolate was examined for morphology and pigmentation. The week of the appearance of colonies was noted. If contamination occurred, the sputum samples were decontaminated again and re-cultured for management of the patient and were excluded from the study. Furthermore, if there was no growth by 8 weeks or if contamination was present, the cultures were discarded and the laboratory forms completed accordingly. In addition to cultural characteristics, Standard Ziehl-Neelsen (ZN) smears were made from characteristic colonies appearing before week 8 to confirm the presence of acid fast bacilli [27], [28], [29].

#### M. tuberculosis growth in liquid broth and microscopic detection

The assay was performed as described previously [8], [11], [14]. Broth cultures were prepared in 24-well tissue-culture plates (Becton, Dickinson and Company, Sparks, MD), broth culture contained Middlebrook 7H9 broth (Becton, Dickinson and Company, Sparks, MD), supplemented with 10% OADC (oleic acid, albumin, dextrose, and catalase) (Becton, Dickinson and Company, Sparks, MD), and PANTA (polymyxin, amphotericin B, nalidixic acid, trimethoprim, and azlocillin) (Becton, Dickinson and Company, Sparks, MD). In each sterile 24-well plate, 1 ml of broth was distributed in each well. For each sample, 2 wells were used to culture the specimen. Each row of the plate was used to test three samples, and 10 samples were run in a single plate. In the remaining four wells, quality controls were incorporated in which two wells were used for the reference strain, ATCC 27294 H37Rv *M. tuberculosis* as a positive control for culture, whereas in the last two wells the broth were left uninoculated as a broth control. One hundred microliters of decontaminated sputum samples was inoculated into two wells for each sample, the same volume was also used for the positive control strain. The plate was then covered with its lid and securely sealed throughout its edge using polyethylene tape (Biorad optical tape, Biorad, Hercules CA, USA) to avoid evaporation and cross-contamination in the plate. The date of inoculation and plate number were recorded on the plate. The dates, plate numbers, and corresponding sample numbers were noted on a plate layout laboratory worksheet prepared for the recording of results. Each plate was incubated at 37°C. To minimize cross-contamination, evaporation and occupational exposure, plates were permanently sealed inside polyethylene ziplock bags after inoculation and were subsequently examined within the bag. The cultures were examined under an inverted light microscope (Hund Wetzlar, Germany) at a magnification of 40× every day from day 4 to day 9, on alternate days from day 11 to day 21 and twice weekly from day 21 to day 40. Positive cultures were identified by cord formation, characteristic of *M. tuberculosis* growth, in liquid medium as described previously [8], [11], [14], [30]. Bacterial and fungal contaminations were recognized by rapid overgrowth and clouding in wells. Non-tuberculous mycobacteria were recognized by their lack of cording or, for *M. chelonae* (which is the only nontuberculous mycobacteria that does form cords), by rapid overgrowth prior to day 5 [7].

### Statistical Data Analysis

Data were entered using EpiData 3.1 (CDC Atlanta, USA) and analyzed using Stata 12 software (College Station, Texas, USA), with the sample as the unit of analysis, to reflect the operational performance of a routine service laboratory. Descriptive categorical variables were summarized as proportions whereas continuous variables were summarized as either means ± standard deviations or medians with interquartile ranges where appropriate. We used a two-sample test of proportions to determine the difference in proportions with their respective confidence intervals (CI). The Wilcoxon Sign Rank test was used to compare the time to culture positivity among the two methods. A p-value of less than 0.05 was used to indicate statistical significance. For calculations of the diagnostic parameters of micro broth culture, such as sensitivity and specificity, we used 2-by-2 contingency tables.

### Cost Estimation for Micro Broth and LJ Cultures

Costs were measured from the health center perspective using the microcosting approach of Drummond et al, in which each component of health care that is used is recorded, and a unit cost is applied to each, as in previous studies [31], [32]. Costs for performing a micro broth and LJ culture included personnel time, laboratory reagents and materials, and equipment; we assumed that equipment had a life expectancy of 10 years, and that 2400 cultures were conducted per year. We assumed straight-line depreciation and a discount rate of 3%. The costs of staff training and the laboratory building, electricity, and other overhead items were excluded because they were considered to be insignificant on a per test basis. Costs were measured in 2011 US$.

## Results

### Demographic Factors and TB Cases

A total of 321 patients with suspected pulmonary tuberculosis were enrolled during the study period and all were included in the analysis. The mean age was 38.7±16.7 years and 195 (60.8%) were males. Of the total number, 164 (51.1%) were HIV positive and 194 (60.4%) were ZN positive. Based on American Thoracic Society diagnostic standards, there were a total of 200 (62.3%) pulmonary tuberculosis cases.

### *M. tuberculosis* Culture Detection, LJ Media and Liquid Broth Assay

Of the pulmonary tuberculosis cases, liquid broth detected 178/200 (89.0% [95% confidence interval [CI], 84.7% to 93.3%]), whereas LJ detected 154/200 (77.0% [95% CI, 71.2% to 82.8%]) (*p*-value = 0.0007). Of the 121 patients without TB, liquid assay correctly identified 116 (95.9%) as negative, and LJ solid assay identified 113 (93.4%) as negative (Table 1). Therefore, the sensitivity of the liquid assay was 89% [95% CI, 84.7% to 93.3%], and the specificity was 95.9% [95% CI, 92.3% to 99.4%]. The sensitivity and specificity of the LJ solid assay was 77% [95% CI, 71.2% to 82.8%] and 93.4% [95% CI, [89.0% to 97.8%], respectively.

**Table 1:** Results of Löwenstein–Jensen solid media and micro broth culture for patients with and without tuberculosis.

Results	Pulmonary tuberculosis cases+	
	Positive	Negative
	N = 200	N = 121
**Solid LJ media** *		
Positive [no. (%)]	154 (77)	0 (0)
Negative [no. (%)]	38 (19)	113 (93.4)
Contaminated [no. (%)]	8 (4)	8 (6.6)
**Liquid broth culture** ×		
Positive [no. (%)]	178 (89)	1 (0.8)
Negative [no. (%)]	19 (9.5)	116 (95.9)
Contaminated [no. (%)]	3 (1.5)	4 (3.3)

+ Pulmonary TB defined by American Thoracic Society.

* Solid Löwenstein–Jensen (LJ) media.

× Liquid broth in microplates with detection by inverted microscope.

### Contamination Rates

Less contamination was observed in the micro broth culture assay than in LJ solid media. Of the 321 liquid cultures, 7 (2.2% [95% CI, 0.6% to 3.8%]) were contaminated. Of the 321 solid cultures, 16 (5.0% [95% CI, 2.6% to 7.4%]) were contaminated (*p*-value = 0.03) (Table 1). There was no growth observed in any sample-free control wells containing only broth in liquid culture, indicating no micro broth culture cross-contamination. In micro broth culture, 100% concordance was found for samples run in duplicate.

### Turn Around Time (TAT) for Micro Broth and LJ Cultures

**Figure 1:**
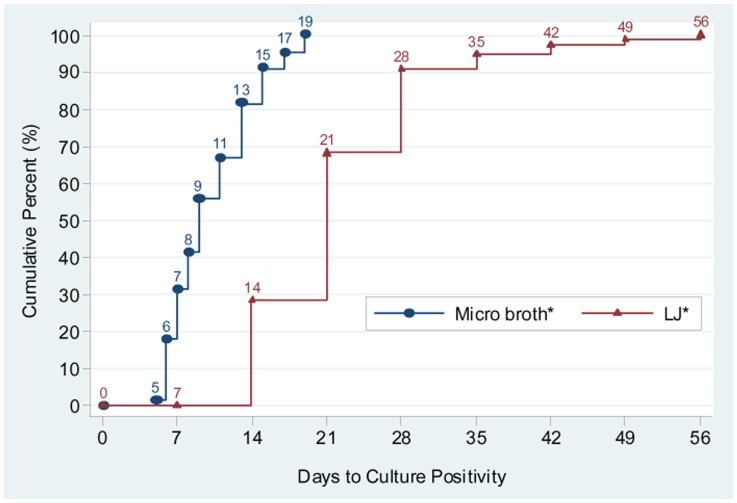
Time to positive culture for solid Löwenstein–Jensen (LJ) versus liquid broth with microscopic detection. *The comparison of time to growth detection for paired 138 samples which were positive for both micro broth culture and LJ culture.

The time elapsed from the date of inoculation to the date of positive-culture result availability for each sample was registered as a turnaround time (TAT). The median TAT for micro broth culture was 9 days, with an interquartile range (IQR) of 7–13 days, whereas for LJ it was 21 days, IQR 14–28 days. The 138 samples that were positive for both micro broth culture and LJ were paired and the time to positive culture results were analyzed. The turn around time to diagnose tuberculosis was significantly shorter for micro broth culture than for the LJ culture (p-value <0.0001) [Figure 1].

### Cost for Micro Broth and LJ Cultures

The cost of performing a micro broth culture was $US 4.56. This included $US 1.06 for laboratory equipment, $US 2.00 for reagents and materials, and $US 1.50 for personnel time. The cost of performing an LJ culture was $US 11.35. This included $US 1.35 for laboratory equipment, $US 5.00 for reagents and materials, and $US 5.00 for personnel time. LJ costs were greater due to the need for equipment for media preparations that the micro broth culture does not require. Furthermore, the labor cost for LJ was higher compared to micro broth culture because it included the cost of preparing the media, inoculation, and reading the media for a longer duration of time than in the micro broth culture.

## Discussion

This study has demonstrated that micro broth culture of *M. tuberculosis* is feasible in underserved settings in Africa, and that it performed better than culture on traditional LJ slants. Our findings indicate that the micro broth technique could improve both patient management and infection control and should be considered for use in resource-limited settings where tuberculosis is endemic.

This study showed that micro broth culture is reliable in detecting *M. tuberculosis* in sputum with greater sensitivity (89% vs. 77%), specificity (95.9% vs. 93.4%) and less time (9 days vs. 21 days) than did LJ. *M. tuberculosis* grows faster in liquid medium than in solid medium and the characteristic cord formation can be visualized microscopically in liquid medium at an early stage and hence favors the detection of mycobacterial growth [11], [12]. Other studies have found similar results, in which *M. tuberculosis* growth in liquid broth is both faster and more sensitive than LJ media [33], [34]. These findings show that micro broth culture is feasible in underserved areas with comparable sensitivity and specificity. Recent studies have shown that resuscitation promoting factors (Rpfs) (proteins produced by *M. tuberculosis* that stimulate mycobacterial growth) can widen the gap between the rate of growth in liquid media and solid media even more by helping neighboring dormant mycobacteria resume replication and grow in liquid broth [35], [36], [37]. The increased growth conditions and the microscopic observation of micro broth cultures permit earlier detection.

The micro broth cultures also have several operational advantages over LJ media. Micro broth culture costs less and has less contamination compared to the LJ media assay [8], [12], [13]. The cost for micro broth culture is lower than that of LJ culture; our study found the micro broth culture to cost US $4.56 per sample, versus US $11.35 per sample for the LJ media assay. However, labor costs are specific to the setting of the study, and the large variability of labor costs will impact the conclusions of cost effectiveness in different settings. Additionally, several studies have shown that the cost for micro broth culture is lower than those of other available culture methods [9], [10], [11] [16]. In the liquid broth assay, 3 positive cultures (1.5%) and 4 negative cultures (3.3%) were contaminated. In the LJ assay, 8 positive cultures (4%) and 8 negative cultures (6.6%) were contaminated.

New technology has begun to revolutionize tuberculosis diagnosis throughout the world. The Xpert MTB/RIF (Cepheid, Sunnyvale, California, USA) test has provided sensitive detection of tuberculosis in a very rapid time frame (3 hours) [38]. That said, this technology requires polymerase chain reaction with sophisticated and expensive equipment. Further, the estimated cost per test, when discounted for High Burden Developing Countries (HBDC), is still $9.98 per sample, not including the cost of the equipment, labor, and maintenance [39]. Additionally, micro broth culture has the advantage of retaining the mycobacteria isolate for further analysis if necessary.

### Conclusion

In conclusion, this study has demonstrated that the liquid assay (micro broth culture) of *M. tuberculosis* is an early, feasible, and inexpensive method for detection of pulmonary tuberculosis in underserved settings in Africa, and performed better than culture on traditional LJ slants.
